# Alterations observed in the interferon α and β signaling pathway in MDD patients are marginally influenced by cis-acting alleles

**DOI:** 10.1038/s41598-020-80374-2

**Published:** 2021-01-12

**Authors:** Chiara Magri, Edoardo Giacopuzzi, Chiara Sacco, Luisella Bocchio-Chiavetto, Alessandra Minelli, Massimo Gennarelli

**Affiliations:** 1 Department of Molecular and Translational Medicine, University of Brescia, Brescia, Italy; 2 Genetics Unit, IRCCS Istituto Centro San Giovanni di Dio Fatebenefratelli, Brescia, Italy; 3 Faculty of Psychology, eCampus University, Novedrate, Como Italy

**Keywords:** Depression, Transcriptomics, Depression, Risk factors

## Abstract

Major depressive disorder (MDD) is a common psychiatric disorder with a multifactorial aetiology determined by the interaction between genetic and environmental risk factors. Pieces of evidence indicate that inflammation and immune activation may contribute to the onset of MDD playing a role in the pathogenetic mechanism. To date, it is not known to which extent the association between MDD and inflammation is shaped by the genetic background or by the presence of environmental factors. To clarify this issue, we analyzed genotype and blood RNA profiles of 463 MDD cases and 459 controls (NIMH-Study 88/Site621) estimating the Genetic and Environmental Regulated eXpression component of gene expression (GReX and EReX respectively). Both components were tested for association with MDD. Many genes belonging to the α/β interferon signaling pathway showed an association between MDD and EReX, only two between MDD and GReX. Also other MDD differentially expressed genes were more influenced by the EReX than by GReX. These results suggest that impact of the genetic background on MDD blood gene expression alterations is much lower than the contribution of environmental factors and almost absent for the genes of the interferon pathway.

## Introduction

Major depressive disorder (MDD) is the leading cause of disability worldwide and is the most common mental health disorder, affecting more than 300 million individuals^1^. MDD has a multifactorial aetiology determined by the interaction of genetic and environmental risk factors^2,3^. Despite its considerable burden, at present the biological mechanisms behind this condition remain elusive. Since the 1950s, several hypotheses have been proposed to explain the molecular mechanisms underlying MDD including the immune inflammation hypothesis. This hypothesis suggests that immune activation, which concurs to inflammation, may contribute to the onset of MDD in at least a subset of cases^4^. Several MDD patients, indeed, show characteristics of a chronic low grade inflammation, including altered peripheral levels of inflammatory cytokines and immune modulators^5–8^. In particular, a large meta-analysis reported increased levels of interleukin-6 (IL-6), tumor necrosis factor (TNF)-alpha, IL-10, soluble IL-2 receptor, C-C chemokine ligand 2, IL-13, IL-18, IL-12, IL-1 receptor antagonist, and soluble TNF receptor 2 in MDD patients compared to healthy controls^9^. The link between inflammation and MDD is also supported by gene expression studies on mRNA transcripts. Although replications of these findings are limited, an up-regulation of pro-inflammatory cytokines, interferons (INFs), transcription factors (NF-kB and CREB1) has been observed in the central nervous system as well as in peripheral blood of MDD patients by many candidate-gene studies^10^. Besides, two largest genome-wide expression studies performed so far on MDD patient blood reported increased mRNA levels of genes in the interferon α/β signaling pathway^11,12^ and a significant enrichment for IL-6-signaling and natural killer cell pathways among genes associated with MDD^12^.

Well known risk factors for MDD and sources of inflammation are chronic psychological stressors and trauma^13^. Many studies reported an association between the inflammatory and immune system gene expression alterations and maladaptive responses to traumatic or psychological chronic-stress^14–17^. Stressful, traumatic life events and more generally environmental risk factors^18–23^ are not the only elements relating inflammation to MDD, indeed twin-based studies have shown a possible effect also of the genetic background^24^. Although with some inconsistencies, many gene-based association studies report a positive association between Single Nucleotide Polymorphisms (SNPs) in genes related to the immunity/inflammatory pathways and MDD vulnerability. A systematic review of these genetic studies is reported in^25^, the most replicated variants include SNPs in IL-1β, IL-6, IL-10, MCP1, TNF-α, CRP, and PLA2 genes.

To date, it is not known to which extent the association between MDD and inflammation is shaped by the genetic background, environmental factors and/or their interaction.

**Figure 1:**
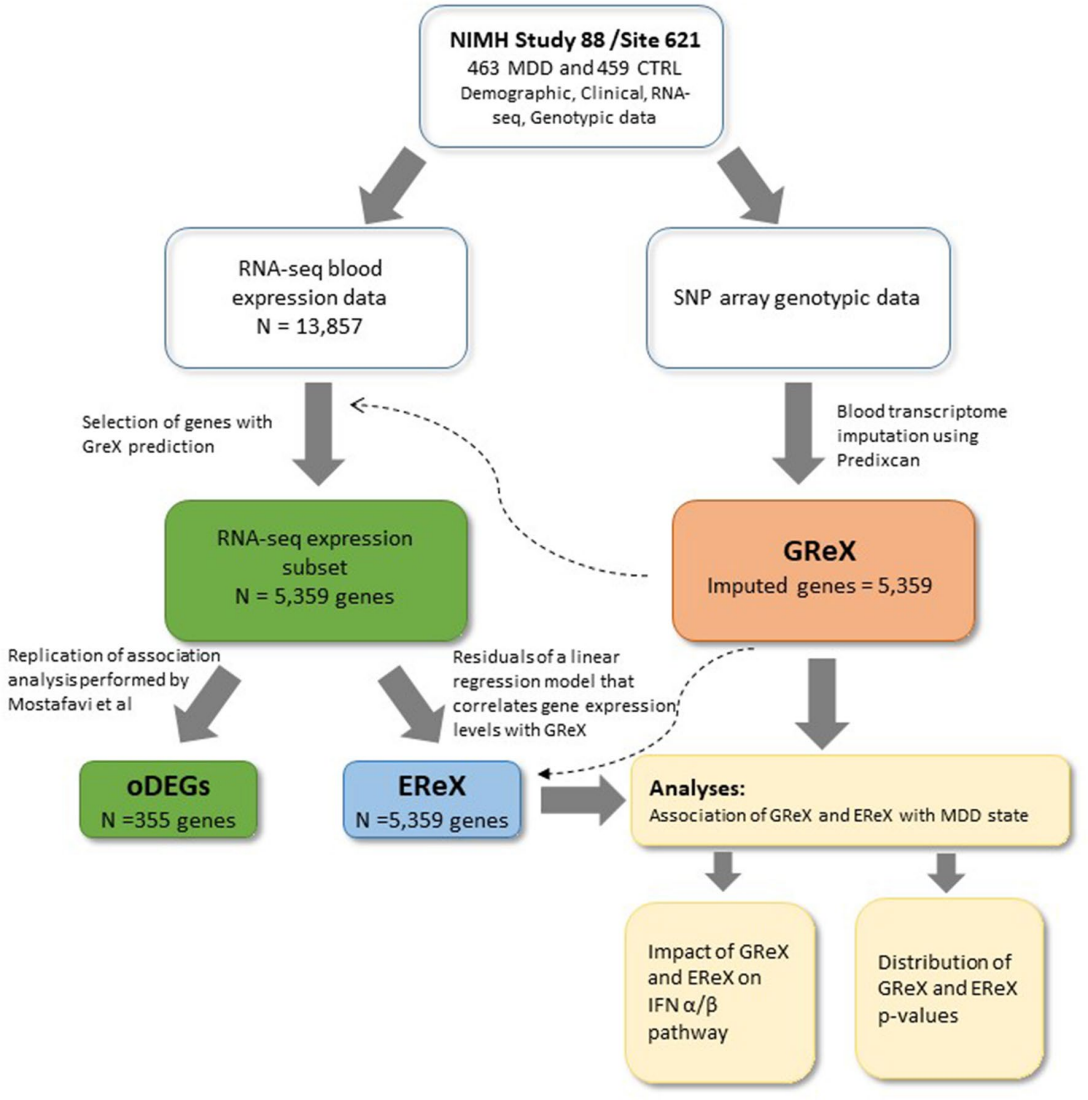
Flowchart of the experimental plan. *GReX* Genetically regulated component of gene expression; *oDEGs* observed differentially expressed genes, that is genes found differentially expressed in our subset of 5359 genes; *EReX* Environmental regulated expression component.

To clarify this issue, we re-analyzed genotypes and blood mRNA expression data of a study including 463 MDD cases and 459 controls (NIMH Study 88/Site621), that previously reported alterations of the inflammatory IFN pathway in the disease^11^. In details, we dissected the expression data of this dataset in two components: the component of gene expression regulated uniquely by cis-acting alleles (eQTL SNP mapping inside 1 Mb of the gene start or end) and that depending on environmental factors. Both components were then tested for association with the MDD phenotype. The experimental plan of the study is graphically summarized in Fig. 1.

## Results

### Performances of PrediXcan predictions in the selected dataset and the reproducibility of original results using the predicted genes subset

Transcriptome imputation methods have become popular methods to predict gene expression from genotypic data. These methods develop prediction models for gene expression starting from a reference data set in which both genome variation and gene expression levels have been measured. In this study, to impute the Genetically Regulated eXpression (GReX) component of blood gene expression, we used the PrediXcan tool^26^. PrediXcan estimates GReX using eQTL SNPs from the GTEx dataset (https://www.gtexportal.org/home/) mapping within ± 1 Mb from the start and end of the genes (here defined as cis-acting alleles). GReX component was predicted for 5359 out of the 13,857 (38.7%) autosomal genes analyzed by Mostafavi and colleagues^11^. Before performing further analyses, we verified the predictive performance of the PrediXcan model in capturing the cis-genetic component of gene expression of our data. We observed a significant positive correlation between cross-validated R^2^ and local estimates of *h*^*2*^. The overall correlation across all genes was 0.77 (*p* < 2.2 × 10^−16^; Supplementary Figure 1). This strong positive relationship confirmed that the PrediXcan model can capture the cis-genetic component of gene expression in the considered dataset.

Furthermore, by enrichment analysis, we verified that the gene subset (N = 5359 genes) was still representative of the original dataset (N = 13,857 genes)^11^: (1) it did not contain an unbalanced representation of some genes categories and (2) it was sufficiently large to detect positive associations with the Interferon pathway. With the only exception of KEGG Lysosome pathway^27,28^, this subset was not enriched for any specific group, confirming the absence of pathway-specific biases compared to the original data set (Supplementary Table 1). Moreover, we observed a good reproducibility comparing the results obtained in our subset with those of the original study for both differential gene expression and gene-set enrichment analysis. Considering the genes reported as differentially expressed (DEGs) in the original paper, with a nominal *p* value < 0.05, 355 of them (oDEGs) were listed in the 5359 predicted genes, including 9 of the top 29 DEGs (defined as FDR < 0.25 in the original paper) (Supplementary Table 2). The pathway analysis performed on these oDEGs was still able to detect the enrichment of the interferon alpha/beta signaling pathway observed in the original paper.

**Table 1:** Observed differentially expressed genes (oDEGs) for the IFN alpha/beta signaling pathway.

Gene	Permutated *p* values			oDEGs Rank
	oDEGs	EReX	GReX	
*OAS1*	1.25E−04	1.25E−04	6.76E−01	1
***MX1***	**2.50E−04**	**2.37E−03**	**1.50E−02**	**2**
*ADAR*	5.00E−04	3.75E−04	5.97E−01	7
***IRF7***	**1.12E−03**	**1.50E−03**	**2.74E−02**	**16**
*ISG15*	1.62E−03	1.62E−03	4.89E−01	20
*IFIT1*	2.00E−03	1.87E−03	8.29E−02	21
*IFI35*	6.88E−03	1.24E−02	1.10E−01	62
*MX2*	1.05E−02	1.01E−02	4.92E−01	88
*OASL*	1.25E−02	2.95E−02	1.54E−01	104
*IFNAR2*	3.84E−02	2.76E−02	9.12E−01	292

Permutation *p* values of oDEGs are reported together with *p* values of association between MDD and EReX and GReX components, respectively. The table shows also gene ranking respect to the observed gene expression among all tested genes. The only two genes with a significant *p* value for the GReX component are reported in bold.

**Table 2:** Enrichment nominal *p* values for association of IFN alpha/beta signaling pathway and MDD.

	N = 30	N = 60	N = 100	N = 150	N = 200
*A. Blood results*					
Observed	2.28E−09	1.76E−07	6.38E−09	7.60E−09	9.46E−08
EDreX	2.28E−09	1.76E−07	1.70E−07	1.58E−07	1.44E−06
GReX	1.00E+00	1.00E+00	3.64E−01	1.44E−01	2.25E−01
*B. GReX brain results*					
Cerebellum	1.00E+00	1.00E+00	1.00E+00	1.00E+00	1.00E+00
Cortex	1.00E+00	1.00E+00	1.00E+00	1.00E+00	1.00E+00
Frontal cortex	1.00E+00	1.00E+00	1.00E+00	1.00E+00	1.00E+00
Hippocampus	1.00E+00	1.00E+00	1.00E+00	1.00E+00	1.00E+00
Hypothalamus	1.00E+00	1.00E+00	1.00E+00	1.00E+00	1.00E+00
Nucl. accu. basal ganglia	1.00E+00	1.00E+00	1.00E+00	1.00E+00	1.00E+00
Putamen basal ganglia	1.00E+00	1.00E+00	1.00E+00	1.00E+00	1.00E+00
Anterior cingulate cortex	1.00E+00	1.00E+00	1.00E+00	1.00E+00	1.00E+00
Caudate basal ganglia	1.00E+00	1.45E−01	2.31E−01	3.28E−01	4.14E−01
Cerebellar hemisphere	1.00E+00	1.00E+00	1.00E+00	4.48E−01	5.50E−01

(A) The analyses were performed on observed expression data, EReX and GReX variables in blood. (B) The analyses were performed on GReX component of the IFN alpha/beta signaling pathway genes in ten different areas of brain.

### Estimation of the Genetically Regulated Expression (GReX) and the Environmental Regulated Expression (EReX) components and their correlation with MDD phenotype

Established that the gene subset was still suitable to detect the association with the interferon pathway, we estimated the GReX and EReX components, and we tested their association with the MDD phenotype for all oDEGs detectable in our dataset.

Of the 64 genes annotated in the IFN alpha/beta signaling pathway in MSigDB v.6.0 (https://www.gsea-msigdb.org/gsea/msigdb/index.jsp), 24 were also listed in our dataset and 10 were detected as DEGs in the original paper. All of them showed a significant association with MDD when the EReX component was considered; only two (*MX1* and *IRF7*) resulted to be associated at a nominal *p* value < 0.05 with MDD when the GReX component was considered (Table 1).

The enrichment analysis performed specifically on this pathway revealed that all tested subsets of 30–200 top genes, ranked by their EReX *p* values, were significantly enriched for genes of the IFN alpha/beta signaling pathway, whereas none enrichment was observed when the analysis was performed on the top gene subsets ranked by their GReX component (Table 2A).

To assess if the GReX component could have a different contribution in brain or in blood expression, we estimated the GreX component of the IFN alpha/beta signaling pathway genes in ten different brain areas, and we tested their association with MDD. In the brain, the mean number of genes of the IFN alpha/beta signaling pathway for whom Predixcan was able to estimate the GReX component was 9.4 (SD = 4.55). Only in caudate basal ganglia and cerebellar hemisphere areas, we observed a nominal significant association between MDD and the GreX component of *IFIT2* (*p* = 0.011) and of *HLA-F* (*p* = 0.032) genes, respectively. Moreover, the enrichment analysis did not reveal any significant enrichment of genes of the IFN alpha/beta signaling pathway among top gene subsets ranked by their GReX component in any of the ten brain areas analysed (Table 2B).

Finally, to test if the contribution of the EReX and GReX components observed for IFN alpha/beta signaling pathway genes could be generalized to other genes, we extended our analysis to all oDEGs.

When we tested the association between EReX component and MDD, we observed a nominal significant *p* value for 280 out of 355 oDEGs. When the same analysis was performed for the GReX component, we observed only 39 genes out of 355 (Table 3). By comparing MDD associated genes for the EReX and GReX components, we observed that the majority of GReX genes (N = 30) did not overlap EReX ones suggesting that the observed differences in expression were mainly driven by the genetic background. Nine genes were associated with MDD for both EReX and GReX components, including 6 genes ranked among the top 30 oDEGs (*MX1*, *RABEPK*, *TNFRSF10B*, *SDK1*, *IRF7*, *RBM6*). Thus, the relatively strong differential expression originally observed between MDD cases and controls for these genes seems due to the combination of the cis-genetic background and the impact of environmental factors and/or clinical variables.

**Table 3:** 39 oDEGs showing a significant association between MDD and GReX component.

Gene	Permutated *p* values			oDEGs rank
	oDEGs	GReX	EReX	
*MX1*	2.50E−04	1.50E−02	**2.38E−03**	2
*TNFRSF10B*	5.00E−04	8.38E−03	**1.93E−02**	3
*RABEPK*	5.00E−04	1.11E−02	**7.13E−03**	4
*SDK1*	7.50E−04	6.25E−04	**2.93E−02**	10
*IRF7*	1.13E−03	2.74E−02	**1.50E−03**	16
*RBM6*	2.25E−03	3.01E−02	**1.96E−02**	25
*ZNF584*	3.88E−03	4.04E−02	**5.61E−02**	35
*EPOR*	4.25E−03	4.33E−02	**1.46E−02**	39
*SCO2*	6.00E−03	1.88E−03	2.58E−01	52
*SGSH*	8.00E−03	2.64E−02	8.99E−02	67
*TSGA10*	9.00E−03	3.04E−02	1.25E−01	74
*XAB2*	9.13E−03	2.81E−02	5.85E−02	78
*ZNF514*	1.05E−02	1.00E−03	3.90E−01	87
*PSMA4*	1.14E−02	4.06E−02	9.06E−02	93
*PLCH2*	1.14E−02	4.73E−02	7.58E−02	94
*TMEM97*	1.21E−02	8.75E−03	3.02E−01	99
*CYP2D6*	1.21E−02	1.45E−02	2.57E−01	100
*AHSA2*	1.29E−02	2.20E−02	2.10E−01	105
*ATPAF2*	1.40E−02	3.50E−03	1.17E−01	113
*ODF3B*	1.50E−02	2.50E−03	1.65E−01	121
*ZNF132*	1.58E−02	3.14E−02	3.43E−01	127
*BLM*	1.68E−02	1.74E−02	3.35E−01	134
*EPS15L1*	1.89E−02	2.15E−02	**4.36E−02**	153
*TMEM121*	1.99E−02	3.46E−02	2.43E−01	159
*UAP1L1*	2.04E−02	2.81E−02	3.22E−01	165
*PEX5*	2.14E−02	3.26E−02	3.40E−01	171
*DNAJC18*	2.40E−02	5.50E−03	3.64E−01	193
*IGSF8*	2.44E−02	1.31E−02	9.94E−02	195
*ZNF354A*	2.58E−02	3.01E−02	2.35E−01	202
*GFRA2*	2.91E−02	1.88E−03	7.00E−01	224
*SLIT1*	3.00E−02	2.09E−02	4.64E−01	233
*ZNF83*	3.16E−02	2.88E−03	4.62E−01	246
*MAN1A1*	3.43E−02	3.96E−04	2.79E−01	261
*CTNNA1*	3.65E−02	2.15E−02	4.71E−01	278
*ANXA6*	3.81E−02	1.50E−03	8.33E−01	290
*HOXA4*	3.95E−02	3.93E−02	8.81E−02	296
*DNASE2*	4.56E−02	2.06E−02	6.34E−01	327
*KIR2DL4*	4.59E−02	1.09E−02	5.08E−02	328
*TERF2*	4.99E−02	2.85E−02	**3.20E−02**	352

Genes with a significant association between MDD and EReX component are highlight in bold.

**Figure 2:**
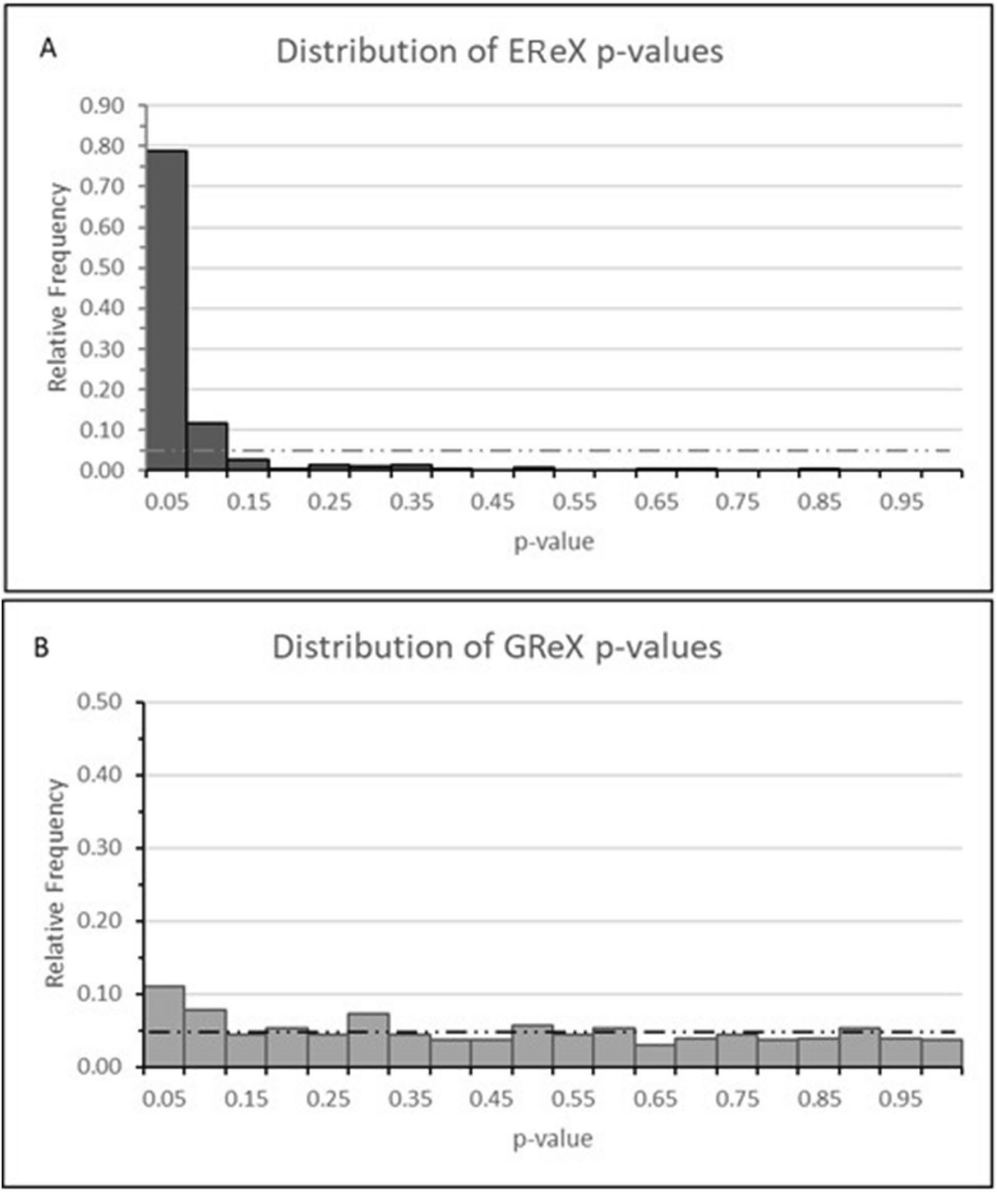
Distribution of nominal *p* values of EReX (**A**) and GReX (**B**) among oDEGs. The figure shows an excess of low *p* values respect to the uniform null distribution (dashed line) in correspondence of both components.

A large excess of genes with small *p* values in the EReX component was observed among oDEGs, whereas the distribution of the GReX *p* values resulted to be substantially flat, distributed uniformly on [0, 1] (Fig. 2). The excess of small *p* values for the GReX component, however, highlighted that the proportion of true positive tests was of Π_1_ = 0.23, indicating a restrained association between differentially expressed genes reported for GReX and oDEGs. These results were supported by the hypergeometric test analysis that revealed a significant over-representation of both genes with GReX or EReX low *p* values among the top 30–300 oDEGs ranked by the association *p* values (Table 4).

**Table 4:** Over-representation of genes with a significant association between MDD and GReX or EReX among top N oDEGs.

	N = 30	N = 60	N = 100	N = 150	N = 200	N = 300
GReX	1.24E−02	1.62E−02	7.80E−04	3.09E−04	1.10E−04	2.97E−04
EReX	1.33E−36	6.65E−68	1.17E−96	1.18E−136	1.78E−175	2.38E-272

Hypergeometric tests were used to evaluate the over-representation of genes showing a significant association between MDD and GReX or MDD and EReX among subsets of N oDEGs, ranked by *p* values of the association test between the observed gene expression and the MDD status, compared to all expressed genes.

## Discussion

The hypothesis that alterations in the immune system regulation can contribute to the onset of MDD had gained increased support in recent years^4^. At present, however, it is not known to which extent the association between MDD and the inflammation pathway is shaped by the genetic susceptibility background, the presence of environmental factors, and/or by their interaction. In order to clarify this issue, we dissected gene expression data of a large genomic/transcriptomic dataset on MDD (463 cases with MDD and 459 controls^11^) in its two components: the Genetically Regulated eXpression component (GReX) and the Environmental Regulated eXpression component (EReX); both components were tested for association with MDD.

GReX component was inferred by Predixcan^26^, a transcriptome imputation method that predicts genes expression from GTEx cis-eQTLs information. EReX component was calculated as residuals of a linear regression model that correlates the observed gene expression levels with the imputed GReX levels.

Genes belonging to the IFN α/β signaling pathway showed a significant association with MDD when the EReX component was considered, whereas only two genes (*MX1* and *IRF7*) resulted to be associated with MDD when the GReX component was taken into account. The altered expression of the interferon α/β signaling genes observed in MDD patients, therefore, seem to be only marginally influenced by cis-acting alleles. This scenario confirms the results of Mostafavi and colleagues showing not significant association between MDD and SNPs within a range of 1 Mb around each interferon gene^11^. Moreover, this observation is in line with one of the largest GWAS performed so far on MDD^2^ that identified only a modest association (false discovery rate q value = 0.039) between immune response genes and the disease susceptibility. A restrained association between genetic variants and the altered IFN pathway expression seems to be a feature not limited to blood. Indeed, the analysis of GReX component estimated in ten different brain areas did not revealed a significant enrichment of genes of the interferon α/β signaling pathway among the top ranked genes: only for two genes (*IFIT2* and *HLA-F*), a nominal association with MDD was observed. These results are in line with a transcriptome-wide association study in the dorsolateral prefrontal cortex of MDD post-mortem brains that identified a set of SNPs in 17 genes correlated to gene expression alteration but none of them was involved in the immune response pathway^29^.

Our results represent an attempt to specifically dissect the contribution of genetic and non-genetic factors on the dysregulation of the inflammation pathway in MDD and support growing evidences showing the role of environmental factors in the IFN/inflammatory alterations in this mental illness. At this regard, our group has recently found an association between IFN pathway gene expression and childhood emotional abuse in a subgroup of MDD patients of the same cohort^30^, suggesting that the inflammatory alterations frequently observed can be partly explained by the high frequency of emotional abuse in these subjects^30^. Another recent study reported increased IL-6 plasma levels, white blood cell count and general inflammation levels in MDD patients with an history of adverse childhood experiences compared to those without^31^. Altered gene expression of the inflammation pathways could likely be driven by exposure to environmental triggers like trauma and physical or psychological stress^32^. Other mediators of the association between MDD and inflammation could be exposure to harmful environmental factors, like airborne pollution and aeroallergens^33^ or diet^23^ and gut microbiota^34^. The use of antidepressants has also been seen to affect the immune system^35^. However, Mostafavi and colleagues in their original paper showed that the association between the IFN alpha/beta signaling pathway and MDD observed in this cohort was not correlated with the use of antidepressant drugs^11^. We tested the correlation between EReX and the use of antidepressant drugs and we found no association (data not shown). Moreover, we could speculate that adverse environmental stimuli can alter the expression and act in depression development mechanisms through epigenetic processes such as DNA methylation, histone modification, microRNA regulation and/or RNA modifications^36–38^. For example, it is well known that the association of childhood trauma with increased inflammation is linked to epigenetic changes in *FKBP5*, a gene implicated in the development of depression^39^. Moreover, a significant hypomethylation of *IL6* gene was reported in association with childhood trauma leading hypothetically to an increased transcriptional gene expression^40^.

In addition, there is evidence that microRNAs are mediators of early life stress vulnerability^41^ and several microRNAs regulate different aspects of the type I IFN signaling pathway, for examples by targeting multiple members of the α/β IFN gene family^41^. Expanding our analysis to all differentially expressed genes, we observed a similar scenario: gene expression differences between MDD cases and controls were mainly due to environmental factors rather than to the cis-genetic background. Indeed, the large majority of oDEGs (79%) showed a nominal significant association with MDD only when it is analyzed for the EReX component. However, despite this strong effect of the EReX component, among oDEGs we observed also some genes showing an effect in the GReX component supporting only for these specific genes, a direct genetic control on differential gene expression observed in MDD. In particular, we identified 39 DEGs that were nominally associated with MDD when the GReX component was considered. For these genes, the differential expression could be partially explained by the effect of MDD risk alleles mapping inside or near the gene. Among these, we highlight the presence of *MAN1A1* (Mannosidase Alpha Class 1A Member 1), *SDK1* (Sidekick Cell Adhesion Molecule 1) and *BLM* (Bloom Syndrome RecQ Like Helicase) that map in loci containing SNPs showing nominal associations with MDD in the recent GWAS^2^. Moreover, *SDK1* genes has recently been associated with depressive symptoms and neuroticism personality trait^42^. We evidence also the presence in the list of *CYP2D6* gene encoding for the cytochrome P450 2D6, one of the most important enzyme involved in the pharmacokinetic of xenobiotics including many antidepressant drugs. Finally, nine of these genes show a positive association with MDD also for the EReX components. They could be particularly relevant as their dysregulation could indicate a synergic effect of cis-genetic and environmental factors.

The weak contribution of the cis-genetic background to gene expression in blood is in line with the findings reported in twin studies that estimated the mean heritability (*h*^*2*^) of blood gene expression ranging between 0.101 and 0.26 depending on the methodology used for gene expression analysis (microarray or RNAseq)^43,44^. The limited effect of GReX component on blood gene expression in MDD patients could in part reflect the fact that MDD is primarily a brain disease^2,3^ as emerged from GWAS studies that consistently reported association of the disease with variants harbored in genes mainly expressed in the brain.

In our study, we used PrediXcan algorithm to impute the genetically-controlled component of gene expression and preliminary quality controls have shown that it captures the cis-genetic component of gene expression pretty well. Indeed, we observed a significant positive correlation between cross-validated R^2^ and local estimates of *h*^2^. However, we are aware that PrediXcan algorithm is based on the analysis of alleles mapping inside 1 Mb of distance from the gene start or end, thus capturing only common genetic variants and cis-acting alleles, while the effect or rare variants and trans-alleles remain undetected. However, according to PrediXcan’s authors, when trans-acting alleles are included in the prediction model the departure from the null distribution was much smaller than the one based on local SNPs^26^. Moreover, the prediction accuracy could differ across genes^45^ and in different populations^46^, and our results may not be immediately generalized for all MDD cases. For this reason, confirmatory studies applying different reference panels including also trans-acting alleles, such as the eQTLgen database^47^, can contribute to clarify the genetic component effect.

As concerns EReX component, since it is calculated from the residuals of a linear regression model that correlates the observed gene expression levels with the imputed GReX levels, it accounts for the effects of environmental factors but it can also include a residual genetic component not captured by GReX (trans-eQTL effects, for example). The impact of trans-eQTL on EReX, however, has been reported to have only a marginal impact on gene expression^41,48^.

In conclusion, the results of the GReX and EReX analyses suggested that the gene expression alterations observed in blood of MDD patients, could be more triggered by environmental factors than by the cis-genetic background. In line with recent studies linking stressful events to MDD^49^, the overall impact of GReX component on gene expression seems much lower than that of EReX and almost absent for genes of the interferon pathways.

## Methods

### Sample

We analyzed clinical and biological data from dataset 7 of National Institute of Mental Health (NIMH) study (Levinson RNA sequencing data from NIMH Study 88/Site 621), encompassing 922 European-ancestry individuals. Data were downloaded from www.nimhgenetics.org, after we have been granted access by data access committee. Sample characteristics were originally described by Mostafavi and collaborators^11^. Briefly, 463 cases with MDD with two or more lifetime episodes or one episode lasting 2 years, and 459 controls who never experienced a 2-week period with depressed mood plus two or more other MDD criteria were included in the database. Subjects were genotyped with the Illumina Omni1-Quad microarray and blood expression levels were obtained through RNA sequencing carried out with an Illumina HiSeq 2000.

### Prediction of Genetically Regulated expression (GReX) component

We applied PrediXcan method using genotypic data of the 922 European-ancestry individuals from Levinson’s dataset to predict GReX. Only SNPs with minor allele frequency (MAF) > 0.05 and in Hardy–Weinberg Equilibrium (Fisher *P* > 0.05) were included in the model. A total of 617,957 SNPs were analyzed using the PrediXcan blood weights matrix based on HapMap SNP set (available from PredictDB). GReX component was estimated for 6590 genes.

To be able to compare GReX estimations with observed expression data, only genes observed with at least 10 reads in at least 100 subjects in the original RNA-seq data were retained. The final dataset was made up of 5359 genes.

Before performing further analyses, we verified on our data the predictive performance of PrediXcan model when capturing the cis-genetic component of gene expression. We analyzed the relation between the predicted and the observed gene expression, by computing tenfold cross-validation R^2^. Moreover, we assessed correlation between cross-validated R^2^ and local estimates of gene heritability (*h*^*2*^). Heritability of gene expression was computed for each gene using mixed-effects models as implemented in GCTA^50^, considering SNPs within 1 Mb from gene boundaries. By enrichment analysis, we verified if the gene set predicted by Predixcan was a representative subset of the data set analyzed in Mostafavi’s paper^11^. At this purpose, the hypergeometric test has been performed on the 1328 canonical pathways from MSigDB v.6.0 to verify concordance of over-represented pathways between our dataset and the full set of 13,857 genes analyzed by Mostafavi and colleagues. Additionally, we verified if our subset was by itself enriched in any of these pathways, to exclude that it contains an unbalanced representation of some genes categories.

### Estimation of EReX variable

EReX variable was obtained from the residuals of a linear regression model that correlates the observed gene expression levels with the imputed GReX levels. Thus, EReX component represents the amount of gene expression variability that is not explained by the cis-genetic component, likely due to environmental factors.

### Association of GReX and EReX components with MDD state

The gene expression analysis was performed following the method described in Mostafavi and collaborators^11^. Likelihood ratio tests (LRTs) have been performed to assess the significance of the association between MDD status and observed gene expression levels, GReX component and EReX component, respectively. The LRT is based on the comparison of the likelihood of the null (background) model, which includes a set of confounding factors with the likelihood of the full model, which includes all the confounding factors of the null model and the gene expression. We considered the 39 confounding factors reported by Mostafavi and collaborators (details are available in original paper^11^, supplementary materials, Table 2): age, sex, Body Mass Index (BMI) and other 21 biological and drug intake variables resulted associated with MDD status or gene expression principal components; the first 10 principal components (PCs) of gene expression and 5 genotype PCs.

The 10 expression PCs were considered as confounding factors for the analysis of EReX, but not for GReX. Indeed, the estimation of the GReX component is based exclusively on the genotype information and therefore the correction for expression PCs was not necessary. Genotype principal components were, instead, included in both analyses, to correct for population stratification.

The *p* values of LRT tests were computed by permutation analyses as proposed by Mostafavi and collaborators^11^. Final *p* values were obtained using permutation analyses (8000 initial permutations or 1,000,000 permutations for three genes with *P* = 0 in the initial 8000). False discovery rate (FDR) was used for multiple hypothesis correction.

### Impact of GReX and EReX on IFN alpha/beta pathway

To explore the role of EReX and GReX components on the significant association observed between MDD and expression of genes involved in interferon alpha/beta signaling, we tested the over-representation of genes belonging to the interferon (IFN) alpha/beta signaling (REACTOME) pathway among genes positive associated (*p* < 0.05) with MDD for the GReX and EReX components, respectively. Of the 64 genes in this pathway annotated in MSigDB v.6.0, 24 were successfully estimated by PrediXcan. As proposed by^11^, the hypergeometric test was used to evaluate the enrichment among the top N genes ranked by association *p* values for the EReX and the GReX component, respectively. Different thresholds of N were chosen (N = 30, 60, 100, 200) to verify the robustness of the enriched signal in the ranked genes.

### Prediction of GReX component in ten brain areas and its impact on IFN alpha/beta pathway

In order to study the impact of brain GReX component of IFN alpha/beta pathway gene on MDD, GreX component was calculated in ten different brain areas: anterior cingulate cortex (acc), caudate basal ganglia (cbg), cerebellar hemisphere (ch), cerebellum (ce), cortex (cx), frontal cortex (fc), hippocampus (hipp), hypothalamus (hypot), nucleus accumbens basal ganglia (nabg) and putamen basal ganglia (pbg). GReX estimation was obtained applying the same criteria reported for blood, but using tissue specific weights matrices available from PredictDB. The number of genes predicted for GreX component was: 2365 (acc); 3252 (cbg); 4003 (ch); 4970 (ce); 3274 (cx); 3067 (fc); 2450 (hipp); 2247 (hypot); 2920 (nabg); and 2577 (pbg).

Association between GReX component of brain and MDD as well as its impact on IFN alpha/beta pathway were tested as previously reported for blood.

### Distribution of GReX and EReX *p* values

To study the impact of the GReX and EReX component on the observed differentially expressed genes (oDEGs), we compared, for these genes, the distribution of the association *p* values computed by LRT in the analyses of EReX and GReX components. We estimated the proportion of true positive association, Π_1,_ assuming a uniform null distribution^51^.

The over-representation of GReX and EReX significant association *p* values among observed DEGs, was computed using the hypergeometric test. The analysis was repeated for 6 different groups of N observed DEGs ranked by association (N = 30, 60, 100, 150, 200, 300).

## Supplementary information

Supplementary Table 1.

Supplementary Information

## Data Availability

The data that support the findings of this study are available from NIMH Center for Collaborative Genomic Studies on Mental Disorders (https://www.nimhgenetics.org/access_data_biomaterial.php) restrictions apply to the availability of these data, which were used under license for the current study, and so are not publicly available. Data are however available from the authors upon reasonable request and with permission of NIMH Center for Collaborative Genomic Studies on Mental Disorders. Weight matrix for transcriptome prediction used during the current study are available in the PredictDB repository (http://predictdb.hakyimlab.org).
