# Supplementary material for: Alterations observed in the interferon α and β signaling pathway in MDD patients are marginally influenced by cis-acting alleles
Source: Sci Rep. 2021 Jan 12;11:727. doi: 10.1038/s41598-020-80374-2 (PMC7804189; doi:10.1038/s41598-020-80374-2)
Supplement: Supplementary file 2 — Supplementary Information [file 41598_2020_80374_MOESM2_ESM.docx]

# Alterations observed in the interferon α and β signaling pathway in MDD patients are marginally influenced by cis-acting alleles

Chiara Magri^1,#,*^, Edoardo Giacopuzzi^2,#^, Chiara Sacco^1^, Luisella Bocchio-Chiavetto^2,3^, Alessandra Minelli^1^, Massimo Gennarelli^1,2^.

**Supplementary Table 2. Top 30 oDEGs in MDD cases.** The top 30 genes found associated with MDD in our subset are listed according to their p-values. The genes that were also among the top 29 genes in the original paper are reported in bold.

| **Gene** | **Heritability** | **Direction** | **Observed Expression** | **Function** |
| --- | --- | --- | --- | --- |
|  |  |  | **Permutation p-values** |  |
| ***OAS1*** | 0.173 | + | 1.25E-04 | Immune function |
| ***MX1*** | 0.120 | + | 2.50E-04 | Immune function |
| *ADAR* | 0.030 | + | 5.00E-04 | RNA editing |
| ***CROCC*** | 0.122 | - | 5.00E-04 | Cell cycle |
| ***H6PD*** | 0.388 | - | 5.00E-04 | ER, converting cortisone to cortisol |
| ***RABEPK*** | 0.182 | + | 5.00E-04 | Vesicle trafficking |
| ***TNFRSF10B*** | 0.422 | + | 5.00E-04 | Apoptosis, immune function |
| ***MAFK*** | 0.077 | - | 6.25E-04 | Transcription activation/acetylation |
| ***NXT1*** | 0.022 | + | 6.25E-04 | RNA binding and transport |
| ***SDK1*** | 0.219 | - | 7.50E-04 | Axon guidance |
| *SH3BP5* | 0.089 | - | 7.50E-04 | Negative regulatory role of BTK-related signaling |
| *MCM9* | 0.175 | + | 8.75E-04 | DNA replication |
| *KIAA1958* | 0.016 | + | 1.00E-03 | Unknown |
| *QRICH1* | 0.025 | + | 1.00E-03 | Inflammation and apoptosi |
| *SNRNP40* | 0.013 | + | 1.00E-03 | Splicing |
| *CD320* | 0.231 | - | 1.12E-03 | Promote B cell differentiation and proliferation |
| *IRF7* | 0.063 | + | 1.12E-03 | Interferon regulatory factor |
| *MSR1* | 0.433 | - | 1.25E-03 | Scavenger receptor activity |
| *DCAF16* | 0.045 | - | 1.62E-03 | Protein binding |
| *ISG15* | 0.064 | + | 1.62E-03 | Innate immune response to viral infection |
| *IFIT1* | 0.016 | + | 2.00E-03 | Interferon-induced antiviral RNA-binding protein |
| *BRD7* | 0.293 | - | 2.13E-03 | May play a role in chromatin remodeling. |
| *SLC2A5* | 0.076 | - | 2.13E-03 | Fructose transporter |
| *TRIM35* | 0.350 | + | 2.13E-03 | Unknown |
| *RBM6* | 0.201 | - | 2.25E-03 | RNA binding protein |
| *SAAL1* | 0.096 | - | 2.37E-03 | Protein coding |
| *XCL2* | 0.466 | + | 2.37E-03 | Innate immune system |
| *TRAT1* | 0.108 | + | 2.63E-03 | Stabilizes the T-cell antigen receptor/CD3 complex at the surface of T-cells |
| *PPM1J* | 0.012 | + | 3.13E-03 | This gene encodes the serine/threonine protein phosphatase |
| *PQLC1* | 0.208 | - | 3.13E-03 | Unknown |


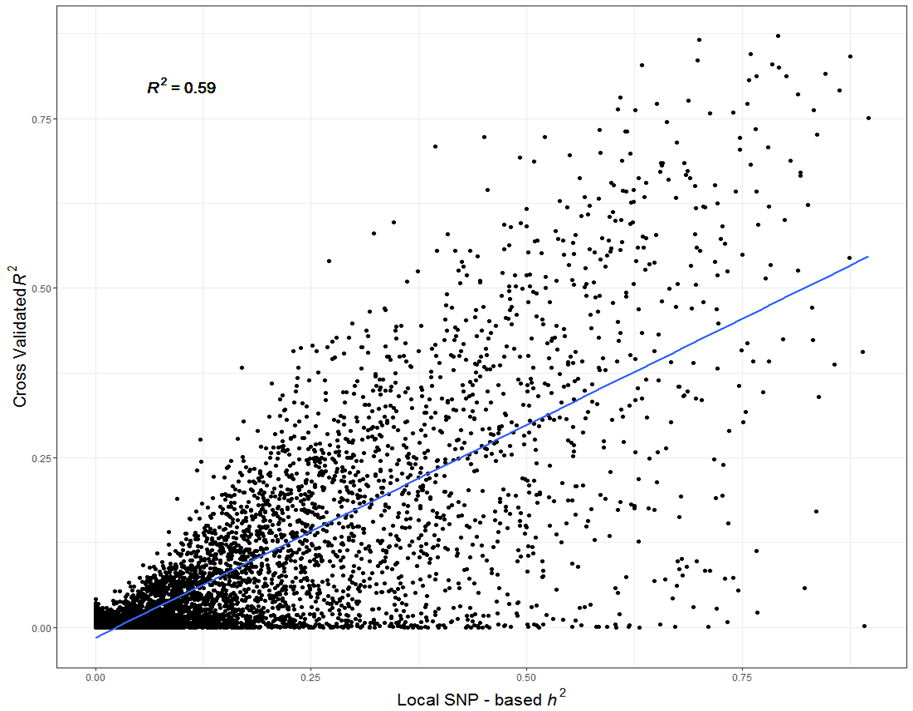


Supplementary Figure 1. Cross-validated R^2^ and local heritability. This figure shows the prediction performance of the PrediXcan tool (cross-validated R^2^ of observed expression versus GReX component) in comparison to gene expression local heritability.
